# A mixed-methods evaluation of a psychosocial intervention to reduce mental health stigma among university students

**DOI:** 10.1007/s44192-026-00551-z

**Published:** 2026-07-29

**Authors:** Josefine Retka, Elena Stoll, Emily Nething, Sara Marie Uhlig, Christin Schwiebert, Samuel Tomczyk

**Affiliations:** 1 Department of Health and Prevention, Institute of Psychology, University of Greifswald, Robert-Blum-Straße 13, 17489 Greifswald, Germany; 2 Department of Psychosocial Medicine, Institute of Medicine Psychology and Sociology, University Medicine of Rostock, Rostock, Germany

**Keywords:** Post-secondary students, Mental health, Stigma, Mental health literacy, Continuum beliefs, Mixed-methods

## Abstract

**Purpose:**

Faced with personal and study-related stressors, students in higher education experience elevated levels of depressive and anxiety symptoms while often showing low help-seeking behavior. Although interventions have shown promise in enhancing student well-being, their potential to address stigma, which is closely linked to help-seeking, remains largely unexplored. The present study evaluates *The Inquiring Mind* (TIM), a Canadian anti-stigma program for post-secondary education, adapted for the German university context, assessing its potential to reduce stigma, increase mental health literacy and encourage continuum beliefs, the understanding of mental health and illness as existing on a spectrum rather than as fixed categories.

**Method:**

A sequential explanatory mixed-methods design was applied, using a quasi-experimental online survey (pre-, post- and follow-up assessment), and a subsequent focus group with program participants. A total of 58 students participated. The mixed-methods approach was selected to gain a better understanding of the program’s impact and the effectiveness of specific components.

**Results:**

Findings revealed significant reductions in stigma in the intervention group at post-test and a significant increase in continuum beliefs at follow-up. While quantitative results showed no improvements in mental health literacy, qualitative analyses revealed greater awareness of mental health issues and self-stigmatizing processes, as well as improvements in the normality aspect of continuum beliefs.

**Discussion:**

Findings support the applicability of TIM in a new cultural context, contributing to research on the emerging concept of continuum beliefs and to the understanding of how interventions can influence post-secondary students’ stigma. This systematic evaluation of an early, campus-based prevention program offers relevant insights for developing prevention strategies addressing students’ post-pandemic mental health needs across diverse cultural and geographic settings.

**Supplementary Information:**

The online version contains supplementary material available at 10.1007/s44192-026-00551-z.

## Introduction

For many post-secondary students, college or university is a complex and often stressful phase of life, characterized by academic, personal, and financial pressures that many students find difficult to navigate. These may include coping with exam-related stress, adjusting to new social environments, or finding a place to live. Such challenges likely play a role in the increased vulnerability to mental health disorders observed in young adulthood [1, 2]. A meta-analysis by Li et al. [3] reports that depression and anxiety symptoms are highly prevalent among college students, with rates of 33.6% and 39.0%, respectively. These conditions are frequently associated with risk factors such as alcohol use, smoking, stress, and sleep problems [3]. Due to the high prevalence of risk factors and potential vulnerability, students in post-secondary education are an important target group of (mental) health promotion and prevention. Notably, mental health impairments are more common among students compared to the general population [4, 5].

Adding to this, a particularly detrimental influence on student mental health was the global COVID-19 pandemic, which led to reduced social interactions and other everyday restrictions. As a result, students commonly reported increased levels of stress and anxiety, as well as reduced quality of life and mood [6–8]. Additionally, increases in alcohol and cannabis use among students were observed, with consumption motives shifting from social and celebratory purposes toward coping with depressive symptoms and boredom [9, 10]. Overall, those disproportionately affected included women, individuals with lower socioeconomic status, limited financial resources, and students in their first year [11, 12].

Although the restrictions have long since been lifted, high levels of psychological distress among university students have persisted beyond the end of the pandemic: Post-pandemic findings indicate that symptoms of anxiety and trauma have not returned to pre-pandemic levels, and that returning to campus has not fully restored students’ mental health [13–15]. Rather, the post-pandemic university environment appears to be characterized by a sustained vulnerability to mental health problems [14].

This persistent psychological burden also has important implications for academic functioning: In Germany, a survey of approximately 180,000 university students found that, among students reporting impairments that substantially interfered with their academic performance, mental health problems were identified as the cause in 65% of cases [5]. These findings underline the considerable need for accessible mental health support and counseling services within university settings, particularly in the post-pandemic context. Providing adequate psychological support may help mitigate mental health problems, enhance academic success, and prepare for future public health crises [13].

However, despite the high level of need, utilization of available support services remains low: Although 95.1% of students with mental health impairments reported academic difficulties [5], and 70.1% were aware of counseling services provided by universities or student unions, only 33.8% had actually used these services. This pattern of low help-seeking behavior is also reflected globally: meta-analyses show that only about one-third of students actively seek help or use available services when experiencing psychological distress [2, 16]. Given the substantial gap between need and service utilization, understanding barriers to help-seeking among university students is essential.

Research indicates that a major barrier to the adequate utilization of support services is poor mental health literacy, along with fears of discrimination and the stigmatization of mental illness [17, 18]. Steinkühler et al. [5] found that 15% of students with physical or mental impairments reported having experienced discrimination in the form of exclusion or degrading treatment for these reasons—an experience that may be associated with increased stress and reduced satisfaction with academic life. On the intrapersonal level, stigma may emerge when individuals internalize culturally shaped negative stereotypes about mental illness, leading to reduced self-esteem and quality of life [19, 20]. At the interpersonal level, stigma can appear through perceived social exclusion, resulting in less openness about symptoms, and at the structural level through institutional barriers such as exclusion from certain professions [19, 20]. Finally, on the public level, stigma reflects societal stereotypes embedded in the broader cultural context [21].

In light of the concerning mental health situation among students and the damaging effects of stigma, there is broad consensus in the literature on the importance of implementing campus-wide initiatives and interventions [17, 22]. Essentially, such programs are encouraged to focus on strengthening resilience, addressing stigma on multiple levels, and facilitating students’ awareness of how they conceptualize and interpret both their own mental health and that of others [17, 22]. Despite increasing attention to student mental health, important conceptual and practical limitations of existing prevention programs remain and are outlined below prior to introducing the present intervention.

Over recent decades, several community-based early intervention models have been developed to address stigma and promote mental health, however, many are not post-secondary-specific [23, 24]. Furthermore, while a number of promising mental health initiatives exist within higher education, such as the *CAMPUS* program in Japan [25] or the *U Bring Change to Mind University* (UBC2M) campaign in the US [26], their efficacy and effectiveness for post-secondary students often remain insufficiently demonstrated [23]. More recently, stepped-care frameworks have been developed worldwide, such as *COMPAS-S* (Australia) [27] and *STAND* (US) [28], an approach that might be particularly promising for integrating mental health prevention and early intervention in higher education contexts [29]. Likewise, in Germany, several cross-university programs, such as *StudiCare* or *Moodgym*, have recently emerged that aim to improve student mental health through minimal stepped-care interventions [30, 31]. However, while these interventions generally encourage openness and acceptance, they do not directly target stigma [30, 31]. Furthermore, these interventions often do not fully utilize the potential of early behavioral and setting-based prevention strategies. They tend to reach students only after (subclinical) impairments have already manifested or have been recognized as such, thus delivering selective or even indicated prevention. Moreover, many of these interventions are disorder-specific, which limits their reach to relatively small and heterogeneous groups, making them less suited to driving broader societal change.

### Current study

To address these gaps, the present study examined *The Inquiring Mind (TIM)*, a Canadian psychosocial intervention for post-secondary students that directly targets stigma using a universal preventive approach. Empirical findings suggest notable improvements in students’ mental health outcomes, including a sustained decrease in stigmatizing attitudes and enhanced resilience evident three months post-intervention [29, 32, 33]. Participants also demonstrated greater willingness to engage in conversations about mental health and to access support services when necessary [29, 32, 33].

In line with current research on effective stigma reduction [24, 34], TIM offers a key advantage by integrating two evidence-based components of successful anti-stigma programs: (1) psychoeducation, which provides background knowledge about mental health concepts and coping resources; and (2) video-based contact with individuals who have lived experience of mental health conditions. Moreover, utilizing not only behavioral but also environmental prevention approaches, TIM includes a module focused on building a healthy campus environment. Thus, it promotes a supportive climate by fostering inclusive language and raising awareness of peer initiatives as well as stigma-reducing institutional practices [29].

Importantly, the intervention extends beyond traditional stigma-reduction approaches by incorporating the mental health continuum, a dimensional framework that has so far seen limited application in student mental health settings. The continuum illustrates that mental health is not fixed but exists on a spectrum, with individuals moving along it at various times in their lives [35].

Continuum beliefs can be conceptualized as a central component of the stigma process and may therefore serve as a cognitive mechanism for stigma reduction by reshaping beliefs about mental health and illness, particularly by reducing notions of “differentness” [36]. Continuum beliefs appear to be multidimensional, encompassing the perception that individuals with mental health problems are fundamentally normal, that symptoms exist along a continuum, and that mental health problems are continuous rather than categorical [37]. As these dimensions are assumed to be modifiable through interventions [37], continuum beliefs may function both as an intervention outcome and a mechanism of stigma reduction. Indeed, not only have continuum beliefs been successfully manipulated by interventions, but also correlational and interventional studies have shown the continuum model to reduce stigma [36, 37].”

Addressing students’ mental health burden and need for effective and accessible prevention, our study extends previous findings by introducing and testing TIM in German higher education for the first time. The adaptation and evaluation of TIM contribute to ongoing efforts to implement effective and accessible mental health prevention in higher education, with a focus on countering stigma and providing early support. We formulated the following hypotheses:

#### Hypothesis 1

Participants in the intervention group will exhibit lower levels of mental illness stigma relative to the control group.

#### Hypothesis 2

Participants in the intervention group will show higher levels of mental health literacy relative to the control group.

#### Hypothesis 3

Participants in the intervention group will hold stronger continuum beliefs relative to the control group.

## Method

### Ethics and deviations from the study protocol

The project is based on the guidelines of Good Scientific Practice of the German Research Foundation and Good Clinical Practice of the German Society for Epidemiology, all human-related procedures were performed in accordance with these guidelines. For the implementation of the empirical studies in the project, approval was obtained from the local ethics committee. Participants provided written informed consent prior to participating in a focus group or interview. For the online survey, informed consent was obtained by agreeing to an online data protection form before starting the survey. All participants were explicitly informed that participation was voluntary and that there would be no negative consequences for choosing not to participate. With regard to data protection, participants were provided with the contact details of the local data protection officer and were informed about how their data would be processed, for example for example in relation to pseudonymization and subsequent anonymization once the three data collection waves had been completed. Participants were also informed of their rights, such as the right to access their personal data or the right to withdraw their consent to the processing of their personal data.

Due to group differences regarding baseline scores of the outcomes and sociodemographic characteristics, we conducted ANCOVAs instead of the planned ANOVAs, controlling for age and baseline scores.

### Trial design, intervention and study objectives

**Fig. 1:**
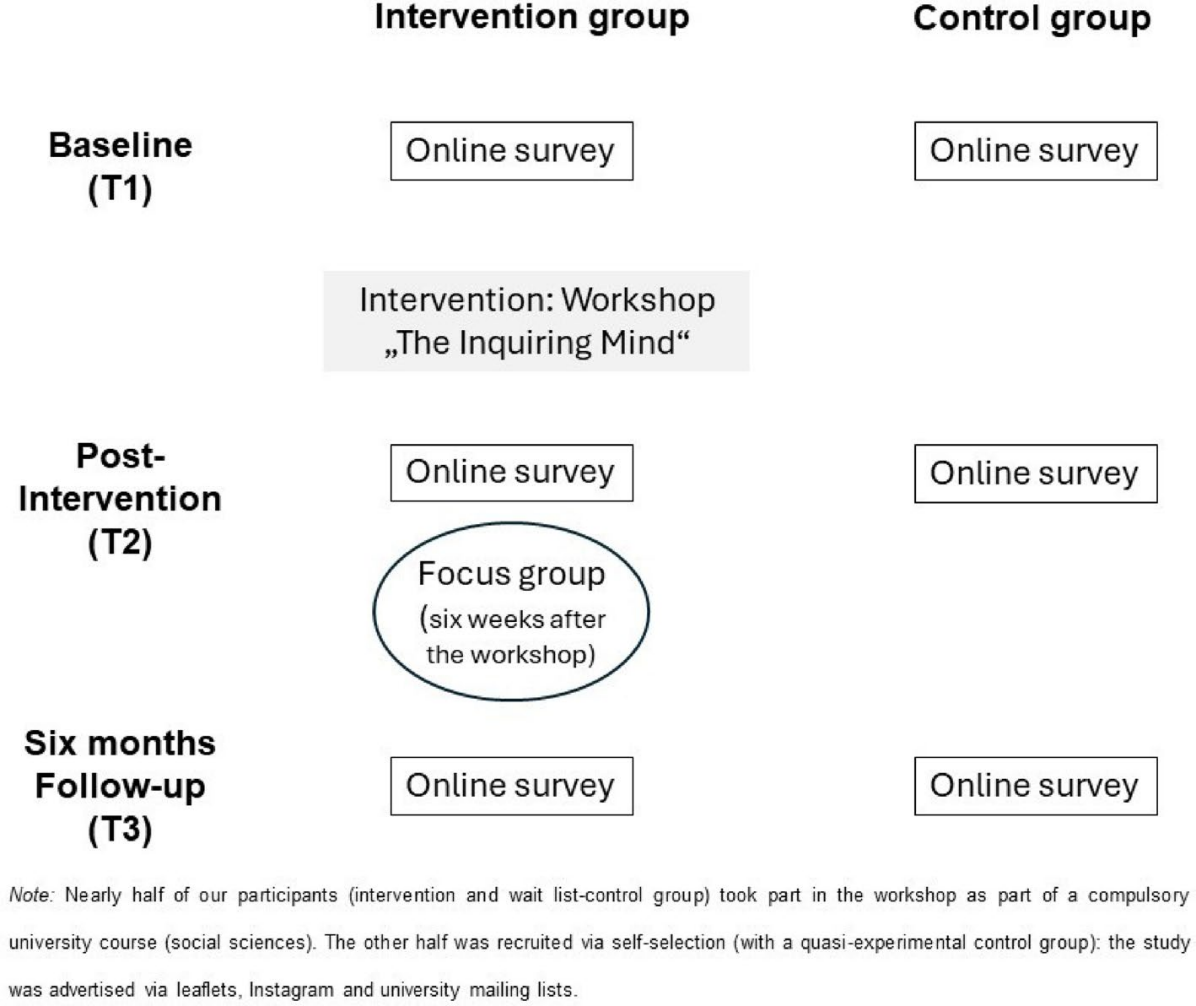
Trial design

To evaluate the effectiveness of TIM within the German higher education context, we applied a sequential explanatory mixed-methods design (QUAN → qual) comprising (quasi-) experimental online surveys and subsequent focus groups, see Fig. 1. Whilst approximately half of the participants were randomly allocated to either the intervention or control group (within a waitlist control design), the remaining participants were recruited through self-selection, as detailed in the recruitment procedure below. Representing the target group of our study, student perspectives played an important role throughout the research process, aligning with GRIPP2 recommendations [38]. Methodological decisions, planning and procedures are described in detail in Nething et al. [39]. Reporting of quantitative results adheres to the TREND statement, while qualitative findings are presented in line with the COREQ checklist [40].

Participants were recruited from February 2024 to November 2024, with last follow-up data acquisition in April 2025. For participation in the workshop, and for each fully completed questionnaire, all participants were offered vouchers worth 10€ each.

Participants were recruited in two different ways: through self-selection and as part of a compulsory course in the social sciences. As regards self-selection, the intervention was included in the university catalogue of health offers, enabling interested students to sign up via this platform. The intervention was also promoted in the university events calendar, via various university mailing lists and Instagram accounts, and leaflets were distributed on campus. Participants in the self-selected control group were acquired via the channels of other German higher education institutions, such as mailing lists. This quasi-experimental design was chosen for pragmatic reasons: The intervention is new and being tested under real-world conditions, therefore, we considered the barrier to entry and wanted to keep it as low as possible to attract greater interest.

The intervention, the workshop *The Inquiring Mind* (TIM) [29], was provided by the third author. Four workshops, each lasting four consecutive hours, were delivered in small face-to-face groups of up to 15 people on the premises of the respective university.

The four-hour workshop comprises four modules, which focus on mental illness and stigma, the Mental Health Continuum Tool, self-care and resilience building, and creating a supportive campus (see Appendix, Table 1 for details). TIM incorporates two evidence-based elements commonly used in anti-stigma interventions [24, 34]: (1) psychoeducational content addressing mental health concepts and coping resources, and (2) video-based contact with individuals with lived experience of mental health conditions. Information was delivered through PowerPoint presentations and supplementary printed materials (e.g., handbook, pocket card).

To adapt the workshop to the German higher education context, the original workshop materials were revised through a co-design process involving students. While the core material, such as the Mental Health Continuum Tool, was considered to be readily transferable to the German context, the different support offers had to be adapted. Further, new contact videos were created in which German students talk about their experiences, whilst retaining the original interview questions.

Testing TIM in the German university context, we expected medium size effects (d = 0.50) [29] regarding the reduction of public mental health stigma (H1), the increase of mental health literacy (H2) and the increase of continuum beliefs (H3).

### Outcomes, data collection, statistical methods and qualitative analysis

Quantitative outcomes are described in Table 1.

**Table 1:** Quantitative outcome measures in the order of hypotheses

Hypothesis	Construct	Questionnaire(s)
(H1)	Mental illness stigma	Public mental illness stigma, context specific: German version of the *Opening Minds Scale—student version* (OMS-WA) [29]: • We translated the OMS-WA into German using a forward-back translation procedure, consulting an expert panel, and pre-testing the scale with the target group; • 23 items, such as “People with a mental illness could snap out of it if they wanted to”; • 5-point Likert scale from 1 = “strongly disagree” to 5 = “strongly agree”; • Internal consistency is good to excellent for the English student version (0.88 ≤ *α* ≤ 0.92) Public mental illness stigma, value sensitive: *Value-based Stigma Inventory* (VASI) [41]: • 15 items, such as “Living together with a mentally ill person restricts one’s own quality of life”; • 5-point Likert scale from 1 = “strongly disagree” to 5 = “strongly agree”; • Good internal consistency (Cronbach’s α = 0.88), and good convergent and construct validity
(H2)	Mental health literacy	German Version of the *Mental Health Literacy Scale* (MHLS) [42, 43]: • 35 items, such as “To what extent do you think it is likely that *Dysthymia* is a disorder”; • 4-point Likert scale from 1 = very unlikely/unhelpful to 4 = very likely/helpful or 5-point Likert scale from 1 = strongly disagree/definitely unwilling to 5 = strongly agree/definitely willing • Good internal consistency for the English version (Cronbach’s α = 0.83), satisfactory construct validity
(H3)	Continuum beliefs	German *Continuum Beliefs Scale* [35] • 9 items, such as “Now and again most of us have symptoms of a mental illness”; • A 5-point Likert scale from 1 = “strongly disagree” to 5 = “strongly agree.” • Satisfactory internal consistency (Cronbach’s α = 0.68), good test–retest reliability, very good factorial and discriminant validity

All outcomes were assessed at T1, T2 and T3

We collected quantitative data through online surveys administered on the SoSci Survey platform [44]. The focus group was conducted by the research team on university premises (second author, a psychotherapist and fifth author, a psychology graduate student with several years of fieldwork experience). Participants were selected via convenience sampling. The interviewers were female, motivated to destigmatize mental illness. Prior to analysis, the focus group was audio-recorded, transcribed with open-access tools, and verified by an undergraduate assistant.

We conducted ANCOVAs following Twisk et al. [45] to compare T2 and T3 scores between the intervention and control groups, controlling for the baseline value of the outcome and relevant sociodemographic covariates (H1–H3), as detailed in the “Baseline equivalence” section. The significance level was set at α = 0.05 for all tests, except for tests of variance homogeneity (part of the assumption checks), where α = 0.10 was used. The effect size reported is β, the standardized regression coefficient, which was chosen to facilitate comparison of adjusted effects across outcomes measured on different scales. In line with Cohen [46], |β| ≥ 0.10 indicates a small effect, |β| ≥ 0.30 a medium effect, and |β| ≥ 0.50 a large effect.

The focus group guide (see Appendix, Table 2) consisted of openly formulated questions regarding changes in mental illness stigma, mental health literacy and continuum beliefs which were discussed in the group context.

We analysed the focus group using qualitative content analysis. An initial deductive coding framework was developed by the second and third authors, drawing on the focus group guide and relevant literature [39]. The entire dataset was coded in MAXQDA version 2022 by the second and the forth author employing a deductive-inductive content-analytic approach as described by Kuckartz and Rädiker [47]. Coding proceeded with independent analyses, followed by consensus discussion. When disagreements persisted, the third and last authors were consulted.

## Results

**Fig. 2:**
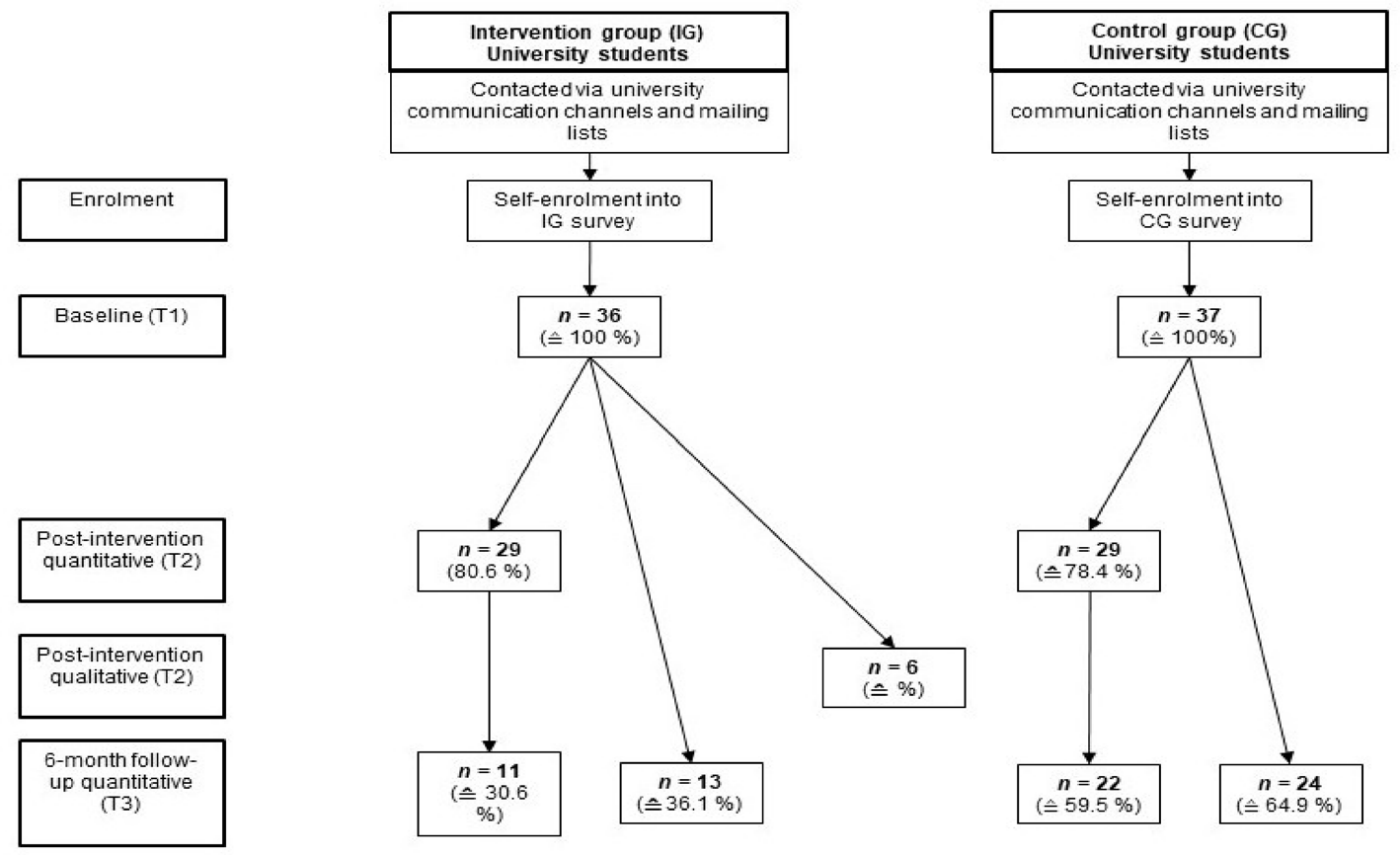
Participant flow

The final sample included only those participants who provided data at baseline and at least one subsequent measurement point (T2 and/or T3). Based on this criterion, the sample was reduced to 31 participants in each group at baseline. The participant flow is presented in Fig. 2.

### Quantitative results

#### Baseline characteristics

Participant demographics are presented in Table 2. No significant sociodemographic differences were observed between groups, apart from age, which approached statistical significance (*p* = 0.06). To account for these differences, age was controlled for in subsequent analyses, based on its correlations with the respective outcomes. Additionally, baseline values of several outcome variables were adjusted for, as intervention and control groups differed significantly in stigma (OMS-WA) and mental health literacy: the control group reported lower stigma and higher mental health literacy (see Table 3). Age and baseline scores were mean-centered in all analyses.

**Table 2:** Participant demographics (baseline values)

	Intervention group			Control group		
Variable	*n*	M	SD	*n*	M	SD
Age	31	21.65	4	31	24	5.83

				Intervention group	Control group
				*n* (%)	*n* (%)
*Gender*					
Female				20 (64.5)	25 (80.6)
Male				8 (25.8)	3 (9.7)
Nonbinary				2 (6.5)	1 (3.2)
Other				1 (3.2)	2 (6.5)
*Education*					
**Postgraduate education**				**2 (6.7)**	**1 (3.6)**
University degree (e.g., Diploma, Master’s, State Examination)				1 (3.2)	1 (3.2)
University of Applied Sciences degree (e.g., Diploma, Master’s)				1 (3.2)	–
**No postgraduate education**				**28 (93.3)**	**27 (96.4)**
Bachelor’s degree (University of Applied Sciences/University)				1 (3.2)	10 (32.3)
General university entrance qualification (A-levels)				26 (83.9)	17 (54.8)
Vocational training (company-based/school-based)				1 (3.2)	–

**Table 3:** Descriptive statistics for the outcome variables by group and time point and ANCOVA results

	Intervention group			Control group			ANCOVA results
Outcome	*n*	M	SD	*n*	M	SD	
*T1*							
MI stigma: OMS-WA (Students)	29	1.85	0.48	30	1.61	0.33	
MI stigma: VASI	29	5.81	1.80	30	5.51	1.19	
Mental health literacy^a^	28	132.96	13.23	30	142.27	7.29	
Continuum beliefs	28	3.84	0.59	30	4.11	0.51	
*T2*							
MI stigma: OMS-WA (Students)^b^	27	1.67	0.37	28	1.71	0.41	*t*(49) = 2.61, *p* = 0.012*
MI stigma: OMS-WA (Students)^b^ × baseline							*t*(48) = 2.86, *p* = 0.006**
MI stigma: VASI^c^	25	5.14	1.36	27	5.61	1.51	*t*(47) = 2.25, *p* = 0.029*
MI stigma: VASI^c^ × baseline							*t*(46) = 3.05, *p* = 0.003**
Mental health literacy^a,b^	25	138.08	9.57	25	139.84	8.07	Ns
Continuum beliefs^b,d^	25	4.08	0.70	25	4.06	0.54	Ns
*T3*							
MI stigma: OMS-WA (Students)^b,d^	12	1.74	0.55	22	1.63	0.30	Ns
MI stigma: VASI ^c^	12	5.28	1.50	21	5.62	1.34	Ns
Mental health literacy^a,b^	12	140.58	8.54	20	141.30	8.20	Ns
Continuum beliefs^b^	12	4.02	0.78	19	4.15	0.59	*t*(25) = − 2.13, *p* = 0.043*

MI stigma = mental illness stigma. All hypotheses were tested using ANCOVAs. Interaction effects are reported for outcomes with violated homogeneity of regression slopes. ^a^Scale reverse-coded to facilitate interpretation. ^b^Adjusted for baseline values and age. ^c^Adjusted for baseline values. ^d^Robust HC3 covariance estimates were used due to violations of homoscedasticity assumptions. †*p* < 0.10. * *p* < 0.05. ** *p* < 0.01.

#### Numbers analyzed

The a priori sample size of *n* ≥ 25 per group as determined by Nething et al. [39] was achieved at T2 but not at T3 (Fig. 1). Attrition analyses were conducted to examine whether participants who dropped out differed from those who completed the study. Specifically, dropout was tested against sociodemographic variables and outcome measures. While no differences were found for sociodemographic characteristics, participants who dropped out reported higher values on both stigma outcomes (OMS-WA: *p* = 0.007; VASI: *p* = 0.022) and were more likely to exhibit lower levels of mental health literacy (*p* = 0.002).

#### Outcomes and estimations

Results for hypotheses H1–3 are summarized in Table 3 (see Appendix, Table A3 for details). ANCOVA assumptions, including linearity, normality of residuals, homoscedasticity, and homogeneity of regression slopes, as well as potential outliers, were assessed. Linearity was confirmed through visual inspection, and quantile-quantile (Q–Q) plots indicated no substantial deviations from normality. In cases of violated homoscedasticity assumptions, heteroscedasticity-robust HC3 covariance estimates were applied. Where the assumption of homogeneity of regression slopes was violated, additional interaction analyses between group and baseline scores were conducted and are reported alongside the main effects. Sensitivity analyses excluding potential outliers yielded comparable results.

##### H1

Mental illness stigma: At T2, significant group differences were observed for stigma outcomes: OMS-WA scores were lower in the intervention group after adjustment for baseline values and age, with a medium effect size (β = 0.45, 95% CI [0.10, 0.80]). However, the assumption of homogeneity of regression slopes was violated, as indicated by a significant interaction between group and baseline OMS-WA scores (β = 0.50, 95% CI [0.15, 0.85]), suggesting that intervention effects varied according to baseline stigma levels. VASI scores were likewise significantly lower in the intervention group after adjustment for baseline values, with a medium effect size (β = 0.41, 95% CI [0.04, 0.77]). Since the assumption of homogeneity of regression slopes was again violated, as indicated by a significant interaction between group and baseline VASI scores (β = 0.56, 95% CI [0.19, 0.93]), the intervention effect appeared to vary depending on participants’ baseline stigma levels.

##### H2

Mental health literacy: No significant group differences were detected at T2, controlling for baseline values and age. The effect size is medium (β = − 0.30, 95% CI [− 0.78, 0.18]).

##### H3

Continuum beliefs: No significant group differences were detected at T2, controlling for baseline values and age. The effect size is small (β = − 0.28, 95% CI [− 0.67, 0.12]).

At the six-month follow-up (T3), no group differences were observed for any outcomes except for continuum beliefs, where the intervention group showed significantly higher scores after adjustment for baseline values and age. The effect size is large (β = − 0.52, 95% CI [− 1.03, − 0.02]).

### Qualitative results

Six intervention group participants took part in the focus group. Focus group participants represented the intervention sample regarding age, and areas of study (law and social sciences). Regarding gender, the focus group participants were even more clearly predominantly female (only one male) in contrast to the intervention sample.

#### H1

Participants reported that they were more aware of their own prejudices or tendency to judge others. This increased awareness for internal stigmatizing processes and an increased understanding for people with mental illness may be indicators for decreased stigmatization:

S06: […] I think that also highlighted that it’s really important that everyone has their own reality. Or their own perception of things. And that these can sometimes be completely different from what you yourself think or perceive in the situation. And that’s why you always question other people’s backgrounds. And yes, what situations might the person be struggling with at that moment, why do they react the way they do? I think the workshop definitely highlighted that. […] (Focus Group_Students, Pos. 45).

#### H2

Most participants were already familiar with mental health issues, but the workshop appeared to deepen or expand this knowledge, for example regarding stigma-sensitive language, potentially contributing to aspects of mental health literacy:

P02: […] Well, we had to deal with such psychological stress a lot during school. Because there were very frequent cases of self-harm in my class. And that’s why we had to deal with it very often. And a lot of it wasn’t NEW to me anymore. But the workshop brought a lot of it back to mind. It had simply been largely forgotten because I hadn’t had any contact with it. And having it brought back to mind was very helpful. Because in the last few weeks, I’ve been thinking more and more about what we’ve learned when everyday situations have arisen or something like that. It was very interesting and also very helpful as a self-reflection, instructive. (…….)

[…]

P01: Yes, well, during my work and travel in […], I also participated in a workshop at […] called “Safe Talk.” There was a relatively large overlap between the things I learned there and the things I had learned here. (.) That’s why there wasn’t a lot/ It was also, of course, very good to reactivate that knowledge. That, um, / But there were also things that I hadn’t learned that then came up here. Especially the language aspect, that was really very relevant to me. (………) (Focus Group_Students, Pos. 26–33).

#### H3

 Regarding continuum beliefs, some participants reported familiarity with the aspect of “Normality of persons with mental health problems”:

P01: Well, that these are just normal people who have problems to deal with [.] (Focus Group_Students, Pos. 37).

P06: […] Because she once told me, (.) yes, um, that she was also in the clinic. But when you were together and had a girls’ night out or something, you never really noticed it. (Focus Group_Students, Pos. 64)

Coding and analysing the focus group transcript we could not find the other two aspects of continuum beliefs (normality of mental health problems, continuity of symptoms) according to Tomczyk et al. [37].

## Discussion

The present study aimed to evaluate the effects of *The Inquring Mind*, an anti-stigma program adapted for the first time to a German university setting, focusing on changes in mental illness stigma, mental health literacy, and continuum beliefs. Quantitative analyses indicated significant reductions in both study-specific and value-based stigma following the program, with medium effect sizes regardless of participants’ age or baseline scores. The results are mirrored in the qualitative data, where participants reported of higher awareness regarding their own stigmatizing attitudes. However, improvements were not sustained at follow-up. No post-intervention group differences emerged for mental health literacy or continuum beliefs. However, the data suggests that the intervention group experienced an increase in continuum beliefs at follow-up. Qualitative findings further pointed to potential changes in mental health literacy and continuum beliefs that may not have been fully captured quantitatively. Notably, the findings also suggest that different dimensions of continuum beliefs may emerge or change at different time points following the intervention. Such novel insights support efforts to develop effective and sustainable stigma prevention in higher education.

### Interpretation

The first hypothesis focused on the impact of the intervention on mental illness stigma. Significant reductions were observed in both stigma outcomes, supporting the effectiveness of the original program as previously evaluated in Canada [29]. These findings are consistent with earlier studies demonstrating the value of psychoeducational and contact-based components in stigma reduction [29, 34] and align with evidence highlighting the relevance of the continuum model as a conceptual basis for reducing stigma [48, 49].

Contrary to expectations, no significant differences in mental health literacy were found, although medium effect sizes were observed. While this contrasts with our qualitative data suggesting greater awareness of mental health issues, this pattern has been observed in previous research. This is examplified in the work undertaken by Reavley et al. [50], which found few improvements in mental health literacy among university students as a result of an intervention adressing mental health literacy, help-seeking, psychological distress and alcohol misuse at a multicampus university in Australia. Consequently, the authors suggested that more personalized or intensive approaches may be necessary to achieve measurable change. Despite these inconclusive findings in the literature, one explanation in the present study may be the relatively low variance and ceiling effects, as baseline values were already high, particularly in the control group, suggesting selection bias. Qualitative data further suggest that most information about mental health issues presented in the workshop was not new to participants, many of whom already reported substantial prior knowledge regarding mental health issues from school or other contexts. Taken together, these findings may indicate that brief universal interventions have limited potential to improve mental health literacy in already high-literacy student populations and that more targeted or adaptive approaches may be needed to achieve measurable change.

Follow-up analyses revealed no sustained effects for stigma or mental health literacy, a result likely explained by high attrition and limited power to detect long-term intervention effects. Similar to mental health literacy, no significant effects emerged for continuum beliefs at post-test, possibly reflecting ceiling effects or limited scale sensitivity in a student group with substantial prior knowledge and awareness. Interestingly, continuum beliefs showed a significant improvement at follow-up. The qualitative data may offer insight into this pattern. As outlined above, continuum beliefs comprise three dimensions [37]. In the post-intervention focus group, participants primarily emphasized the normality of people with mental health problems, whereas the other dimensions (continuity of symptoms and normality of mental health problems) were mentioned less frequently.

This pattern may indicate that some dimensions of continuum beliefs may be more readily influenced by interventions than others. For example, recognizing the continuity of symptoms may require greater personal experience or reflection over time, whereas perceiving people with mental health problems as “normal” may be more easily formed from a distance, for example through videos presented during the intervention. These interpretations remain tentative but may help explain why changes across dimensions of continuum beliefs could emerge at different time points following the intervention, highlighting a novel consideration for stigma-focused interventions. Notably, previous research has rarely differentiated these dimensions as distinctly [36, 37], typically measuring only general continuum beliefs or continuity of symptom experience [37, 51]. The present study thus adds to the conceptual understanding of continuum beliefs and highlights the importance of considering multidimensionality in future research.

### Limitations

A key limitation of this study is the small sample size and substantial attrition at follow-up; conclusions should therefore be drawn with caution. Furthermore, the non-randomized quasi-experimental design may have introduced selection effects and (unmeasured) group differences, thereby limiting internal validity and causal inference despite statistical adjustment. Voluntary participation may have introduced self-selection bias, as students with greater interest in mental health topics and higher levels of mental health literacy may have been more likely to participate. A possible explanation for the lower stigma scores observed in the control group appears speculative: it could be that the control group occurred to have lower values by chance, or that participation in a survey addressing changes in mental well-being attracted students who were already particularly open about mental health issues. Randomized studies are needed to further explore and support the findings of this study.

In addition, participants who dropped out had significantly higher baseline stigma scores and lower mental health literacy than those who completed follow-up assessments. This suggests that the follow-up sample may have been systematically biased toward participants with lower stigma and higher mental health literacy, thereby limiting the generalizability of the findings to the original sample. Additionally, the assumption of homogeneity of regression slopes was violated for OMS-WA and VASI at T2, indicating that intervention effects varied as a function of baseline levels. This limits the interpretation of ANCOVA-adjusted group effects, as these represent average effects across participants and may mask stronger or weaker effects depending on initial stigma levels.

### Implications for future research and practice

Future studies should examine whether stable effects for TIM at follow-up can be observed for stigma when using larger and more diverse samples. Furthermore, Pinto-Foltz et al. [52] reported that significant differences in mental health literacy between the intervention and control groups emerged only several weeks after the intervention. As a delayed effect at follow-up was observed for continuum beliefs in our study, and both constructs are of relevance in interventions to reduce stigmatizing attitudes [29, 36, 37, 53], future research could examine potential shared mechanisms between mental health literacy and continuum beliefs, as well as factors that might explain why these effects may emerge gradually over time. Moreover, a promising next step for research is to further develop the theoretical concept of continuum beliefs by clarifying its dimensions and mechanisms and to examine their effects within post-secondary populations, as most existing studies have focused on general population samples [37]. To support longer-term intervention effects, future interventions could benefit from more explicitly integrating continuum-based activities, such as asking students to reflect on how stress, emotions, and well-being fluctuate over time in themselves and others. Additional adjustments, may include booster sessions [29], extended follow-up intervals, or take-home reflection materials to account for possible delayed effects.

Additionally, systematic assessment of non-response and reasons for non-participation would be valuable, particularly with regard to underrepresented groups such as male students and vulnerable populations (e.g., those with lower economic status). To strengthen reach and engagement, embedding anti-stigma programs within university curricula may represent a promising strategy, as relying solely on voluntary participation often fails to engage those most in need [23]. At the same time, future interventions should further adapt to the realities of contemporary student life by addressing barriers such as time demands, scheduling constraints, and location.

In light of the persistent post-pandemic mental health burden among university students, future research should further explore accessible and broadly implementable strategies for stigma-reduction interventions. In particular, digital and mobile-based delivery formats may be well suited to contemporary higher education settings, where students increasingly rely on flexible and hybrid forms of support [29]. By offering accessible, low-threshold, and non-judgmental forms of support independent of time, mobility, or location, such approaches may help overcome practical and stigma-related barriers to help-seeking. Existing stepped-care programs such as StudiCare, Moodgym, and institution-specific initiatives like me@JGU have already demonstrated effectiveness in promoting student mental health [12, 30, 31]. Extending such digital approaches to stigma-reduction interventions may therefore represent a promising direction for future prevention efforts in higher education.

Additionally, participant-oriented approaches that actively engage students in the development and evaluation of intervention programs may be a promising strategy for increasing program effectiveness [54]. Such involvement has been reported by students as empowering [54]. Similarly, peer-led approaches such as OpenMinds [55] indicate that student participation in program delivery may hold potential for increasing program acceptance in students. Finally, in line with previous research emphasizing the importance of engaging diverse stakeholders for effective implementation of global mental health interventions, strengthening collaboration between university leadership, student representatives, and campus mental health services may contribute to the long-term sustainability of anti-stigma programs [56].

## Conclusion

In sum, *The Inquiring Mind* demonstrated a reduction in stigmatizing attitudes in its first adaptation in a different cultural context. In particular, the findings make a novel contribution to stigma-focused intervention research by highlighting the importance of considering different dimensions of continuum beliefs and how they may respond differently over time. Qualitative findings further revealed multiple individual-level changes in students’ awareness, perceptions, and knowledge regarding their own mental health and that of others. In light of persistent post-pandemic mental health challenges, reaching students, particularly vulnerable subgroups, is of high relevance. While the results require replication in larger samples, this study provides a valuable foundation for future research aimed at tailoring interventions more effectively and exploring accessible digital implementation formats to further strengthen the reach and impact of stigma-reduction efforts in higher education.

## Supplementary Information

Below is the link to the electronic supplementary material.

Supplementary Material 1

Supplementary Material 2

Supplementary Material 3

## Data Availability

The preregistered and peer-reviewed study protocol, study materials, and research data are available at the OSF: https://osf.io/qrjce/?view_only=562269481229499c9467d750b7021e4c.

## Declarations

### Clinical Trial Register

The trial was preregistered in the German Clinical Trials Register (trial ID: DRKS00033523) on 31/01/24 and has also been transferred to the WHO registry (main ID: DRKS00033523).
