# Supplementary material for: A mixed-methods evaluation of a psychosocial intervention to reduce mental health stigma among university students
Source: Discov Ment Health. 2026 Jul 29;6(1):140. doi: 10.1007/s44192-026-00551-z (PMC13421715; doi:10.1007/s44192-026-00551-z)
Supplement: Supplementary file 1 — Supplementary Material 1 [file 44192_2026_551_MOESM1_ESM.docx]

**Manuscript:**

**Reducing mental health stigma on campus: a mixed-methods evaluation of a psychosocial setting-based intervention for German university students**

Supplementary Table 1: Overview of the workshop modules

| **Module number and name** | **Module content** |
| --- | --- |
| **1 Mental Health and Stigma** | - Basic concepts of mental health and illness - Definitions - Mental Health Continuum - Types and effects of stigma - Knowledge of how to reduce stigma and other barriers at the campus - Person-centered language - Overcoming barriers for seeking help |
| **2 Mental Health Continuum** | - Mental health fact and prevalences for German students - Recognize changes in your own mental health and take appropriate measures - Applications of the Mental Health Continuum Model - Changes in substance use and addictive behaviours - Seeking help |
| **3 Self-care and building resilience** | - Recognize and practice coping strategies - External and internal stressors - Stress reactions (fight, flight, freeze) - Resilience - Big 4-strategies (deep breathing, positive self-talk, mental replay, goal setting) |
| **4 Creating a supportive campus** | - Suicide prevention - Recognize and use available resources to protect yourself and others - Support and help services - Recognize and use available tools and resources to create a healthy campus |
