# Supplementary material for: A mixed-methods evaluation of a psychosocial intervention to reduce mental health stigma among university students
Source: Discov Ment Health. 2026 Jul 29;6(1):140. doi: 10.1007/s44192-026-00551-z (PMC13421715; doi:10.1007/s44192-026-00551-z)
Supplement: Supplementary file 2 — Supplementary Material 2 [file 44192_2026_551_MOESM2_ESM.docx]

**Manuscript:**

# Reducing mental health stigma in young adults: a mixed-methods evaluation of a psychosocial setting-based intervention for university students

Authors: Josefine Retka^a^ (ORCID-ID: 0009-0005-3508-2581), Elena Stoll^a^* (ORCID-ID: 0009-0009-8661-2166), Emily Nething^a^ (ORCID ID: 0009-0009-8800-069X), Sara Marie Uhlig^a^ (ORCID-ID: 0009-0006-4580-5365), Christin Schwiebert^a^ (ORCID-ID: 0009-0005-7643-3157) & Samuel Tomczyk^a^ (ORCID ID: 0000-0002-2846-5489)

^a^Institute of Psychology, Department of Health and Prevention, University of Greifswald, Greifswald, Germany

* Corresponding author: Elena Stoll, e-mail: [elena.stoll@uni-greifswald.de](mailto:elena.stoll@uni-greifswald.de); University of Greifswald, Institute of Psychology, Department of Health and Prevention, Robert-Blum-Straße 13, 17489 Greifswald;

**Table A3:** **Quantitative results for the hypothesized outcomes (H1-3)**

| **Outcome** | *b* | *SE* | β | 95% *CI* | *t* | *df_t_* | *p* |
| --- | --- | --- | --- | --- | --- | --- | --- |
| **T2** | | | | | | | |
| MI stigma: OMS-WA (Students) ^a^ | .17 | .06 | .45 | [.04, .30] | 2.61 | 49 | .012* |
| MI stigma: VASI ^a^ | .57 | .25 | .41 | [.06, 1.08] | 2.25 | 47 | .029* |
| Mental health literacy ^c^ | -2.70 | 2.12 | -.41 | [-6.97, 1.57] | -1.27 | 44 | .210 |
| Continuum beliefs | -.17 | .12 | -.28 | [-.42, .08] | -1.40 | 45 | .168 |
| **T3** | | | | | | | |
| MI stigma: OMS-WA (Students) ^a^ | -.03 | .10 | -.09 | [-.24, .17] | -.34 | 30 | .734 |
| MI stigma: VASI ^a^ | -09 | .30 | .07 | [-.53, .71] | .30 | 29 | .767 |
| Mental health literacy ^c^ | -2.98 | 2.41 | -.36 | [-7.93, 1.96] | -1.24 | 26 | .226 |
| Continuum beliefs | -.35 | .17 | -.52 | [-.69, -.01] | -2.13 | 25 | .043* |

*Note.* MI stigma = mental illness stigma. All hypotheses were tested using ANCOVAs. The intervention group constitutes the ANCOVA reference category, i.e., positive values for *b* imply that control group participants score higher on the respective outcome measure, whereas the opposite is true for negative values of *b*. All *b*s are adjusted for baseline differences on the respective outcome measure.

^a^ After controlling for age. ^b^ Scale reverse-coded to facilitate interpretation.

† *p* < .10. * *p* < .05. ** *p* < .01.
