# Supplementary material for: A mixed-methods evaluation of a psychosocial intervention to reduce mental health stigma among university students
Source: Discov Ment Health. 2026 Jul 29;6(1):140. doi: 10.1007/s44192-026-00551-z (PMC13421715; doi:10.1007/s44192-026-00551-z)
Supplement: Supplementary file 3 — Supplementary Material 3 [file 44192_2026_551_MOESM3_ESM.docx]

**Manuscript:**

**Reducing mental health stigma on campus: a mixed-methods evaluation of a psychosocial setting-based intervention for German university students**

Supplementary Table 2: Focus group guide, original German version translated for publication

**BEFORE THE FOCUS GROUPS: PREPARATION FOR FACILITATORS**

**Please bring the following materials to the focus groups:**

- *Printed guide (this document)*
- *Recording device (e.g., smartphone)*
- *Notepaper and pens → for personal notes, for participants*
- *Participant consent forms (2 per participant) → 1 for research team, 1 for the participant*
- *List of values (1 per participant) → for the participant*
- *Vouchers*
- *Voucher receipt form → for research team*

**FOCUS GROUP PROCEDURE**

**0) Arrival & introduction (15 minutes)**

Allow a brief settling-in

Welcome participants

Ask participants to complete consent forms (2 copies)

Present the aim of the focus group by saying:

“The aim of this focus group is to evaluate the workshop ‘The Inquiring Mind”; the topics or questions include:

- Your impressions of the workshop
- To what extent did you learn something new / has your view of psychological conditions (at campus) changed?
- Is there anything you have done differently since the workshop? Any challenges?
- What did you like best and what are your suggestions for future sessions?”

Then present the agenda and group rules by saying: “Our plan for today is …

- to have a discussion round of about 1.5 hours,
- there will be introductory questions on the various topics,
- the group discusses these; a range of responses is explicitly welcome and there are no ‘wrong’ answers,
- the facilitators will often ask in-depth follow-up questions,
- some questions may need to be answered in writing,
- notes may be taken during the focus group,
- the discussion will be recorded, then anonymised in writing, and the file will be deleted afterwards.
- The group rules for the focus group are the same as in the workshop:
  - what is discussed in the group stays in the group,
  - the group is a non-judgemental space,
  - respectful and courteous interaction is important.”

Then finish by asking participants: “Do you have any questions or comments?”

Clarify potential questions and then announce that the recording will be started:
“OK, then I will start the recording now!”

START THE RECORDING

*Note to facilitator: Unless otherwise stated, the following section is structured as follows: a main question is presented, followed by sub-questions. Optional questions, additional prompts or notes to the facilitator are printed in italics.*

**1) Reaction (5 minutes)**
How did you experience the workshop?

- How did you feel during the workshop?
- How did you feel immediately after the workshop?
- What thoughts or emotional processes did the workshop trigger in you?
- Which aspects/elements of the workshop did you find particularly positive?
  - … regarding the content of the workshop
  - … regarding the group interaction
  - … regarding the facilitation

What motivated you to attend the workshop?
 *Note to facilitator: If there is no reaction: Do a go-round in which each participant is asked to briefly share their experiences.*

**2) Learning (40 minutes)**
What new things have you experienced/learned from the workshop? With regards to … (5 minutes)

- … handling stress, ways to promote your mental health?
- … mental health conditions, especially on campus; information about support services, especially on campus?
- *Note to facilitator: If nothing has changed: what was the reason?*

To what extent has the workshop altered or reinforced your attitude towards (people with) mental health conditions, especially on campus? (10 minutes)

- *Note to facilitator: If there is no reaction, ask: Do you feel or think differently about (people with) mental health conditions now?*

If the workshop has changed or reinforced your attitude towards (people with) mental health conditions, especially on campus**:** (10 minutes)
*Note to facilitator: It is important here that any change or reinforcement in the factors below is related to the workshop; if necessary, remind participants of this connection.*

What role does/do …:

- … newly acquired knowledge about mental health play?
- … the concept of a continuum of mental health play?
- … the idea that people with mental health conditions are more than their condition play?
- … empathy for people with mental health conditions play?
  - *Note to facilitator: If there are few answers or very global, ask: Can you describe more precisely how your empathy shows?*
- …. reduced fear of contact with people with mental health conditions play?

Are there any other thoughts or emotional processes that have contributed to a change in your attitude?

To what extent has the workshop changed your attitude towards support services? (5 minutes)

- *Note to facilitator: If there is no reaction, ask: Do you feel or think differently about support services now?*

To what extent has the workshop supported values from this overview, in your view? (10 minutes)

- *Note to facilitator: After a short discussion, provide the list of values; additional values may be suggested.*
- To what extent did any of the workshop content conflict with values that are important to you in life?

**3) Behaviour (10 minutes)**
Is there anything you have started to do differently since the workshop, specifically on campus? With regards to …

- … dealing with stress?
- … dealing with other people who experience psychological strain?
- … or is there anything else?
- *Note to facilitator: If nothing has changed, ask: what was the reason?*

Are the workshop’s ideas applicable to your everyday life – to your studies and to your personal environment?

- If yes, how will you apply these ideas?
- If no, what obstacles exist?
- *Note to facilitator: If there is no reaction, ask: What further input would you have liked to help you handle these obstacles better?*

**4) Final question(s) aimed at suggestions for improvement** (10 minutes)
For the final part of the workshop, please now list your top 3 workshop components on the slips of paper in front of you.

*Note to facilitator: The following two questions can be discussed with the group again or also answered in writing if time is tight.*
Is there anything else you would have liked to take away from the workshop?
What would you have changed in the workshop design?

- *Note to facilitator: If there is no reaction, specify: With regard to the content, facilitation, and group exchange*

**5) End** (10 minutes)
*Note to facilitator: Provide a summary of the topics discussed, including participants’ impressions of the workshop, what they have learned, possible changes in attitudes and behaviours, suggestions for improving the workshop*

*End the session by* *thanking the participants for their input, handing out vouchers, and having them complete forms confirming voucher receipt. Take photos of the forms afterwards.*
